# Analysis of factors of willingness to adopt intelligent construction technology in highway construction enterprises

**DOI:** 10.1038/s41598-023-46241-6

**Published:** 2023-11-07

**Authors:** Zhi-chao Zhou, Yi-kun Su, Zhi-zhe Zheng, Yi-lin Wang

**Affiliations:** College of Civil Engineering and Transportation, Northeast Forestry University, Harbin , 150040 Heilongjiang China

**Keywords:** Engineering, Civil engineering

## Abstract

This study aims to investigate the factors that influence the willingness of highway construction enterprises in China to adopt intelligent construction technology. Based on the existing literature, a TOSE framework was proposed, and four dimensions and 15 hypothesized influencing factors were identified through expert interviews. By using a combination of PLS-SEM and ANN, 513 survey data were analyzed to determine the linear and non-linear relationships of the influencing factors on the willingness to adopt. The results showed that all 14 hypothesized factors had varying degrees of positive or negative effects on the willingness to adopt, except for organizational culture, which was found to have no significant impact. Specifically, technology cost was found to be the most influential negative factor, while market demand and organizational structure were the most influential positive factors. The findings of this study have important reference value for decision makers and participants in highway construction enterprises, as well as other construction companies when considering the adoption of smart construction technologies. The originality of this research lies in the novel application of the TOSE framework to investigate smart construction technology adoption, and the combined use of PLS-SEM and ANN to examine both linear and nonlinear relationships between variables for the first time.

## Introduction

With the continuous development of technology and economy, intelligent construction technology has gradually been widely applied. Smart construction technology refers to the application of information technology, automation technology, robotics and other technologies in building engineering design and construction processes. Examples include building information modeling (BIM), intelligent robots, and unmanned aerial vehicle (UAV) monitoring^1^. As a new construction concept, intelligent construction technology is widely used in civil engineering due to its advantages of high efficiency, resource conservation, low cost, sustainability, and easy operability. Especially in highway construction, intelligent construction technology can achieve high engineering quality and efficiency, and reduce a large number of manual operations and time-consuming processes^2^. In this context, more and more highway construction enterprises are beginning to apply intelligent construction technology in their production and management. However, although intelligent construction technology has received widespread attention and recognition, there are still some difficulties and challenges in its application in the field of highway construction^3^. Intelligent construction technology is an important technical support to ensure the quality and efficiency of highway construction. Researching corporate adoption willingness can help related enterprises to better apply these technologies and enhance core competitiveness by realizing corporate technological transformation and management upgrading, enabling enterprises to formulate more scientific technical strategies. Meanwhile, research results can provide basis for the government to formulate more reasonable and effective policies to support corporate technological application, and directly offer theoretical basis and data support for corporate and governmental decision-making. Therefore, this paper will conduct research on the current adoption willingness of intelligent construction technologies and its influencing factors in highway construction enterprises, and put forward future research directions.

In recent years, many scholars have conducted research on the willingness and influencing factors of adopting intelligent construction technology. Zhou et al. analyzed the driving factors for the adoption of intelligent construction technology in highway construction enterprises using the fuzzy DEMATEL-ISM method, providing important reference for decision-makers and participants in highway enterprises^3^. Through a qualitative review of the research clusters on blockchain in intelligent construction technology, Liu et al. revealed profound insights into research challenges and gaps. Hwang et al. investigated the current application status of intelligent construction technology in the construction industry and developed a data-driven roadmap to promote the intelligent transformation of the construction industry^4^. Jiang et al. established a mediation effect model to study the impact of intelligent urban construction on GTFP and GDP^1^. Ameyaw et al. explored the driving factors that professionals and industry leaders pay attention to in order to ensure the successful adoption of smart contracts for economically efficient delivery of construction projects^5^. Ahmadisheykhsarmast et al. proposed a new approach to blockchain-based smart contracts, creating a secure, trustworthy, and transparent bidding environment^6^.

**Figure 1:**
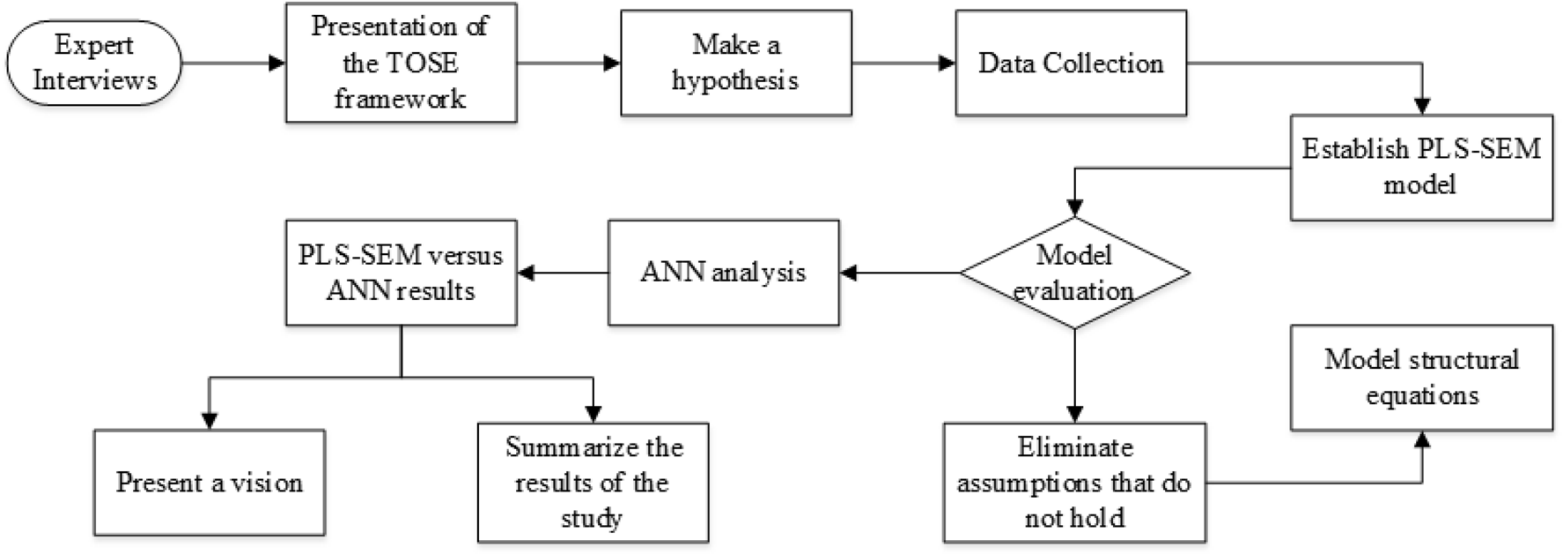
Mind map.

Although many scholars have studied the willingness and related factors of highway construction enterprises adopting intelligent construction technology, existing research lacks systematicity and comprehensiveness. Based on a review and summary of existing research, this paper analyzes the willingness and influencing factors of highway construction enterprises adopting intelligent construction technology, as well as future research directions, in order to assist highway construction enterprises in better applying intelligent construction technology, improving work efficiency and quality, and promoting the development of highway construction.Specifically, based on the TOSE framework, this study systematically examines the impacts of technological, organizational, environmental, and social factors on firms' adoption willingness of smart construction technologies. Through questionnaire survey, PLS-SEM and ANN statistical analysis methods, it clarifies the mechanisms, importance, and priorities of various factors, providing theoretical basis and empirical support for enterprises to formulate scientific and rational adoption strategies of smart construction technologies. This study can make up for the deficiencies of current research and broaden the academic horizons in the adoption of smart construction technologies.To provide a clear and structured perspective, the following is a mind map showing the analysis process and its key steps, which will help readers better understand our research methods and workflow, as shown in Fig. 1.

## Influencing factor assumptions

In order to analyze the influencing factors of highway construction enterprises' willingness to adopt intelligent construction technologies, it is necessary to comprehensively consider all possible influencing factors. Firstly, this chapter constructs a research framework. Then, through open interviews, it widely solicits opinions from industry experts and scholars, summarizes and induces possible influencing factors of adoption willingness. Finally, it proposes hypotheses based on the collected influencing factors, so that empirical research can be conducted in the following text to verify or negate them.

### Expert interviews

To ensure the reliability and authenticity of our research findings, we contacted 64 professionals, experts, and scholars in the industry, ultimately gaining recognition from 42 of them. Through open-ended interviews lasting no less than 15 min each, we collected and improved the factors that influence the willingness of Chinese highway construction enterprises to adopt intelligent construction technology^7^. Unlike traditional survey methods, open-ended interviews can better understand the opinions and experiences of experts and scholars, and obtain more detailed information. Specific expert background information is shown in Table 1. Each interview record was summarized according to four steps: reading and rereading records, identifying influencing factors, reviewing influencing factors, and determining influencing factors, leading to the identification of 15 important influencing factors.

**Table 1:** Background information of experts.

Features	Features	Number of people
Educational background	Undergraduate	7
	Masters	12
	PhD	23
Relevant work experience	5–10 years	9
	10–15 years	16
	More than 15 years	17
Work unit	Constructor	8
	Designer	14
	Higher education institutions	20
Jobs	Technical positions	9
	Management positions	15
	Technical + management positions	18
Title	Intermediate title	17
	Senior title	25

This study expands the Technology-Organization-Environment (TOE) framework by incorporating social environment factors, thus forming the Technology-Organization-Society-Environment (TOSE) framework. Specifically, the adoption of intelligent construction technologies by highway construction enterprises can be analyzed from the following four aspects when applying the TOES framework: technological context, including the availability, complexity, and other attributes of the relevant technologies; organizational context, including the enterprise's scale, structure, resources and other internal factors; environmental context, including competitive environment, policies and regulations, suppliers and other external environmental factors; social context as a new addition, including socio-cultural perceptions, public attitudes and other social environmental factors. These factors directly or indirectly influence technology adoption^3^. It categorizes the factors influencing technology adoption into three dimensions: Technology, Organization, and Environment. In the literature on the adoption of new technologies, many scholars have chosen the TOE framework as the basis for studying the adoption and application of intelligent construction technology^8^. The application of the TOE framework enables enterprises or governments to better understand and evaluate their own advantages and limitations in adopting intelligent construction technology, thereby improving their willingness and success rate of adoption^9^.

Compared to the TOE framework, the TOSE framework is more comprehensive as it adds the dimension of Society to the dimensions of Technology, Organization, and Environment. This includes, but is not limited to, internal organizational factors such as organizational structure, human resources, and management systems, which directly or indirectly influence technology adoption^10^. Socioeconomic factors such as income level, education level, and cultural background also directly or indirectly affect technology adoption. Social factors consider the attitudes of employees towards technology adoption, the level of organizational support, and the contributions of technology adoption to society, thus providing a better explanation of the acceptance of new technology in society . Therefore, the TOSE framework is of significant importance for understanding the decision-making process and influencing factors of enterprise adoption of intelligent construction technology, and it can provide theoretical support for enterprises to make informed adoption decisions. The following are the four-dimensional hypotheses proposed in this study based on the TOSE framework, focusing on the factors influencing the willingness of highway construction enterprises to adopt intelligent construction technology.

### Hypotheses on the technical dimension

Based on the previous research findings, we have identified a large number of influencing factors in the technological dimension. After sorting and summarizing, we have obtained a comprehensive review result as shown in Table 2. When selecting technologies, enterprises need to conduct a comprehensive evaluation based on factors such as technological feasibility and economic viability^11^. In order to promote industrial upgrading, governments and enterprises need to fully assess the technological feasibility and strengthen support in terms of technological assistance and training to enhance the willingness and effectiveness of technological innovation adoption^12^. The literature review shows that the technological advantages of intelligent construction technology have a promoting effect on the popularization of digital twins and the promotion of BIM technology^13^. Olawumi et al. concluded that reducing technological complexity and optimizing project objectives are key driving factors for implementing intelligent sustainable practices in the construction industry^14^. Taking BIM as an example, despite significant progress in its technological openness, it has not been fully adopted, and its ultimate advantages have not been fully utilized by industry stakeholders. The lack of widespread adoption of BIM seems to be related to risks and challenges that may hinder its effectiveness^15^. Based on research on smart contracts related to blockchain, although their application increases the cost of network construction, they ensure information security in terms of user privacy, system control layer, and market transactions^16^. Therefore, this study proposes the following hypotheses:

#### H1a:

 Technological feasibility has a significant positive effect on adoption willingness;

#### H1b:

Technological advantage has a significant positive effect on adoption willingness;

#### H1c: 

Technological complexity has a significant negative effect on adoption willingness;

#### H1d:

 Technological risk has a significant negative effect on adoption willingness;

#### H1a:

 H1e: Technological cost has a significant negative effect on adoption willingness.

**Table 2:** Technical dimension influencing factors of Chinese highway construction enterprises ' willingness to adopt intelligent construction technology.

Influencing factors	Description	References
Technical feasibility	Technical feasibility refers to the practicality of new technology in actual application, including its maturity, stability, reliability, and so on. This factor has a direct impact on the willingness of enterprises to adopt new technology	^11,12,17^
Technological advantage	Technological advantage refers to the actual benefits that new technology can bring to a company, such as cost reduction and efficiency improvement. This factor also has a direct impact on the willingness of enterprises to adopt new technology	^13,18–21^
Technological complexity	Technological complexity refers to the implementation difficulty of new technology, including the technological knowledge requirements and human resource demands. This factor also has a direct impact on the willingness of enterprises to adopt new technology	^14,19,22–24^
Technological risk	Technological risk refers to the risks that new technology may face during implementation and application, such as data security and system instability. This factor also has a direct impact on the willingness of enterprises to adopt new technology	^15,17,25^
Technological cost	Technological cost refers to the cost expenditures that a company needs to invest in order to introduce new technology, including equipment purchase, personnel training, system integration, and so on. This factor also has a direct impact on the willingness of enterprises to adopt new technology	^16,19,20,26,27^

### Organizational factors hypothesis

The understanding and recognition of intelligent construction technology within the organization of highway construction enterprises can greatly promote the adoption of intelligent construction technology, including organizational culture, organizational structure, management support, and employee participation, as shown in Table 3.

**Table 3:** Organizational dimensional influencing factors of highways construction enterprises' willingness to adopt intelligent construction technology.

Influencing factors	Description	References
Organizational culture	Organizational culture refers to the common characteristics of employees' values, beliefs, behavior patterns, and other aspects within the enterprise. Previous studies have shown that it has a significant impact on willingness to adopt intelligent construction technology	^29,33–35^
Organizational structure	Organizational structure refers to the characteristics of internal division of labor, communication, and collaboration within the enterprise. A flatter organizational structure usually supports innovation and change more, and helps to promote the adoption of intelligent construction technology	^10,30,36,37^
Management support	Management support refers to the attitude and level of support of senior management towards the adoption of intelligent construction technology. If the senior management holds a positive attitude and support for intelligent construction technology, then internal employees are more inclined to adopt these technologies	^19,31,38^
Employee engagement	Employee engagement refers to the role played by internal employees in the adoption process of intelligent construction technology. A higher level of employee participation can promote the understanding and recognition of intelligent construction technology within the enterprise, thus promoting its adoption	^32,39,40^

The maturity of safety culture is crucial for preventing unsafe behaviors, especially in industries with high injury and fatality rates such as civil construction^28^. Surveys have found that the Spanish construction industry recognizes the benefits of knowledge management, but systematic knowledge management has not been widely implemented. The survey results clearly indicate that organizational culture change is essential for successful knowledge management^29^. Research has shown that organizational structure plays an important role in smart city governance^30^. Taking the example of Internet of Things (IoT) technology, it can reduce operating costs, facilitate construction site management, and enhance the efficiency of managers^31^. Employee involvement in the process of adopting new products can provide enterprises with more creativity and suggestions, thus promoting the adoption and dissemination of technology^32^. Therefore, this study proposes the following hypotheses:

#### H2a: 

Organizational culture has a significant positive impact on willingness to adopt;

#### H2b: 

Organizational structure has a significant positive impact on willingness to adopt;

#### H2c: 

Management support has a significant positive impact on willingness to adopt;

#### H2d:

Employee participation has a significant positive impact on willingness to adopt.

### Environmental dimensional influencing factors hypothesis

The influencing factors of the environmental dimension can be mainly categorized into local environment, competitive environment, and economic environment, as shown in Table 4. Research by Yuan et al. demonstrated that enterprises that originally held a negative attitude towards BIM adoption can become positive under government subsidy policies, which proves the occurrence of BIM technology diffusion^41^. Wong et al. pointed out that market dynamics and competitive pressure significantly influence the behavioral intention of Malaysian small and medium enterprises to adopt blockchain operations and supply chain management^19^. Asadi et al. examined the factors of green innovation and their potential impact on hotel performance. By examining 183 hotels in Malaysia, they found that the environmental and economic performance factors have the greatest influence on the adoption of green innovation^37^. Therefore, this study proposes the following hypotheses:

#### H3a:

 The local environment has a significant positive impact on willingness to adopt technology;

#### H3b:

The market environment has a significant positive impact on willingness to adopt technology;

#### H3c: 

The economic environment has a significant positive impact on willingness to adopt technology.

**Table 4:** Environmental dimensional influencing factors of highways construction enterprises' willingness to adopt intelligent construction technology.

Influencing Factors	Description	References
Local environment	Local environmental factors refer to the policy guidance and support provided by the government at the macro level for a certain area, which plays a key role in the technological innovation and development of enterprises in that field	^41–45^
Market environment	Market Environment factors refer to the market competition environment in which a product or service is located, as well as factors such as consumer demand and preferences for that product or service	^44,46–48^
Economic environment	Economic environmental factors refer to the relevant institutions, policies, and other factors that affect the economic development and operation of a country or region	^37,49–51^

### Hypotheses regarding social dimensional factors

The influencing factors of the social dimension can be mainly categorized into market demand, social cognition, and cultural differences, as shown in Table 5. Research by Du et al. found that market demand has a positive influence on the adoption of low-carbon buildings (LCB), emphasizing the importance of considering low-carbon synergistic benefits^52^. Fatima et al. discovered that cognition has a significant impact on the willingness to adopt environmental-friendly technologies (EFT)^53^. Lee et al. found that four cultural factors, namely uncertainty avoidance, individualism, contextuality, and time perception, have a significant influence on users' perception of mobile internet services after adoption^54^. Therefore, this study proposes the following hypotheses:

#### H4a: 

Market demand has a significantly positive impact on the willingness to adopt;

#### H4b:

 Social cognition has a significantly positive impact on the willingness to adopt;

#### H4c: 

Cultural differences have a significantly negative impact on the willingness to adopt.

**Table 5:** Social dimensional factors affecting the adoption of intelligent construction technology by high-speed road construction enterprises.

Influencing factors	Description	References
Market demand	Market demand refers to the degree of demand for a technology in the market. In the field of high-speed road construction, with the development of technology and the increase in construction speed, the demand for intelligent construction technology in the market continues to grow	^52,55,56^
Social perception	Social perception refers to the social recognition of the technology. When intelligent construction technology first emerged, it was met with skepticism and rejection by some people because the technology was relatively unfamiliar. However, as the technology is promoted and used, people's understanding of intelligent construction technology gradually deepens, and its acceptance also increases	^36,53,57,58^
Cultural differences	Cultural differences refer to differences in cultural traditions, values, and behavioral norms among different regions, ethnic groups, and countries. These differences may have an impact on the adoption of intelligent construction technology, and different cultural values may lead to different technology choices	^54,59–61^

In conclusion, this paper proposes an adoption intention analysis framework for intelligent construction technology in high-speed road construction enterprises based on the TOSE theoretical framework. This framework includes 15 key influencing factors in four dimensions: technology, organization, environment, and society. The development of this framework is based on discussions with industry practitioners, experts, and scholars. Table 6 is presented to summarize the factors that affect the adoption intention of intelligent construction technology in high-speed road construction enterprises in China, which can be used to analyze the driving mechanism of the adoption intention of intelligent construction technology.

**Table 6:** Summarizes the factors affecting the adoption intention.

Dimension	Number	Influencing factor	Dimension	Number	Influencing factor
Technical dimension	H1a	Technical feasibility(TF)		H2d	Employee engagement(EE)
	H1b	Technological advantage(TA)	Environmental dimension	H3a	Local environment(LE)
	H1c	Technological complexity(TC)		H3b	Market environment(ME)
	H1d	Technological risk(TR)		H3c	Economic environment(EE`)
	H1e	Technological cost(TC`)	Social dimension	H4a	Market demand(MD)
Organizational dimension	H2a	Organizational culture(OC)		H4b	Social Perception(SP)
	H2b	Organizational structure(OS)		H4c	Cultural differences(CD)
	H2c	Management support(MS)			

## Methodology

Traditional research methods have predominantly relied on single qualitative or quantitative approaches, analyzing data through texts, statistical data, or oral communication data. However, with the increasing diversity and complexity of research backgrounds and topics, mixed methods have gradually become a mainstream research approach^62^. By combining different types of data and applying various analytical methods, mixed methods can yield more comprehensive and accurate research results^63^.

Partial Least Squares Structural Equation Modeling (PLS-SEM) is a statistical analysis method commonly used for structural equation modeling with small sample sizes and highly collinear data. It aims at prediction by estimating parameters of structural equation models using the partial least squares algorithm. PLS-SEM can obtain stable component and path coefficient estimates with small sample sizes, and effectively handle situations where high collinearity exists among measured variables. In recent years, PLS-SEM has been widely applied in management research, marketing research and other fields, owing to its advantages in small sample models and high-dimensional complex models^64^. In terms of predictive performance, PLS-SEM can be comparable to other predictive modeling methods and is widely applicable to practical prediction problems^65^. Compared to Composite-Based Structural Equation Modeling (CB-SEM), PLS-SEM has higher accuracy, better discriminant validity, and the ability to handle unconventional data types^66^.

However, PLS-SEM can only analyze linear relationships. Therefore, this study combines it with Artificial Neural Networks (ANN) to examine both linear and non-linear relationships and their importance in adoption intention. An artificial neural network (ANN) is a computational model that mimics the structure and operational principles of biological neural networks. It consists of a large number of interconnected neural units that can realize highly nonlinear mapping and approximate arbitrarily complex functions through learning from training data. ANN can be used for various tasks, such as nonlinear modeling and processing high-dimensional data, adaptive learning and black-box methods^67^. Thus, in this study, the first step involves applying PLS-SEM to analyze the intention model and hypotheses of intelligent construction technology adoption among highway construction enterprises in China. The second step is to employ ANN to ensure that the complexity of the model is not oversimplified by PLS-SEM.

### Data collection

The survey questionnaire research method is now widely used in the construction field because of its high scientificity and convenient operability. Based on the survey questionnaire method, this study collected survey data from industry practitioners, experts, and scholars to distinguish and verify the determinants of China's highway construction enterprises' adoption intention of intelligent construction technology.

The design of this survey questionnaire mainly includes: title, explanatory instructions, basic information of the survey subjects, subjective test questions, and concluding remarks. The survey questionnaire is shown in Appendix 1. All subjective test questions are based on the Likert five-point scale. The collection of survey questionnaires lasted for three months, with a total of 644 distributed and 546 returned questionnaires. After excluding cases with answer time less than five minutes or incomplete/fake responses, 513 valid survey data were finally obtained. According to industry data, there are about 38,321 highway construction enterprises in China, with over 1 million employees. Government management departments and experts and scholars have about 100,000 people. Combining the enterprise scale and regional distribution, stratified random sampling method is used to send questionnaire links to enterprises, government departments, and institutions of higher learning of different scales and regions to collect samples. According to the sample size estimation method, with 95% confidence level and 5% error precision, the required minimum random sample size is 385. This study issued 644 questionnaires and recovered 513 valid questionnaires, meeting the requirements. The representativeness of various groups is relatively strong, and the data distribution is reasonable. The basic information of the survey object is shown in Table 7.

**Table 7:** Experts' background information.

Features	Type	Number of people
Educational background	Bachelor's degree	89
	Master's degree	285
	Doctorate and post-doctorate	139
Relevant work experience	5–10 years	94
	10–15 years	256
	Over 15 years	163
Employer	Design institutes	123
	Construction enterprises	262
	Higher education institutions	128
Position	General manager	37
	Project manager	122
	Chief engineer	74
	Department manager	163
	Professor and associate professor	117

Tests show that the KMO measure is 0.871, greater than 0.8, which verifies the moderate sample size and correlation of variables. The Crobach α coefficient is 0.892, greater than 0.8, which fully demonstrates that the scale has good internal consistency reliability. This further verifies the scientific nature of the samples and data indicators and lays a reliable foundation for follow-up studies. Specific reliability and validity analysis results are shown in Table 8.

**Table 8:** KMO and Bartlett’s test.

Projects		Test value
KMO	Metrics	0.871
Bartlett’s test for sphericity projects	Approximate cardinality	2184.256
	Df	120
	Sig	0

### PLS-SEM model estimation

**Figure 2:**
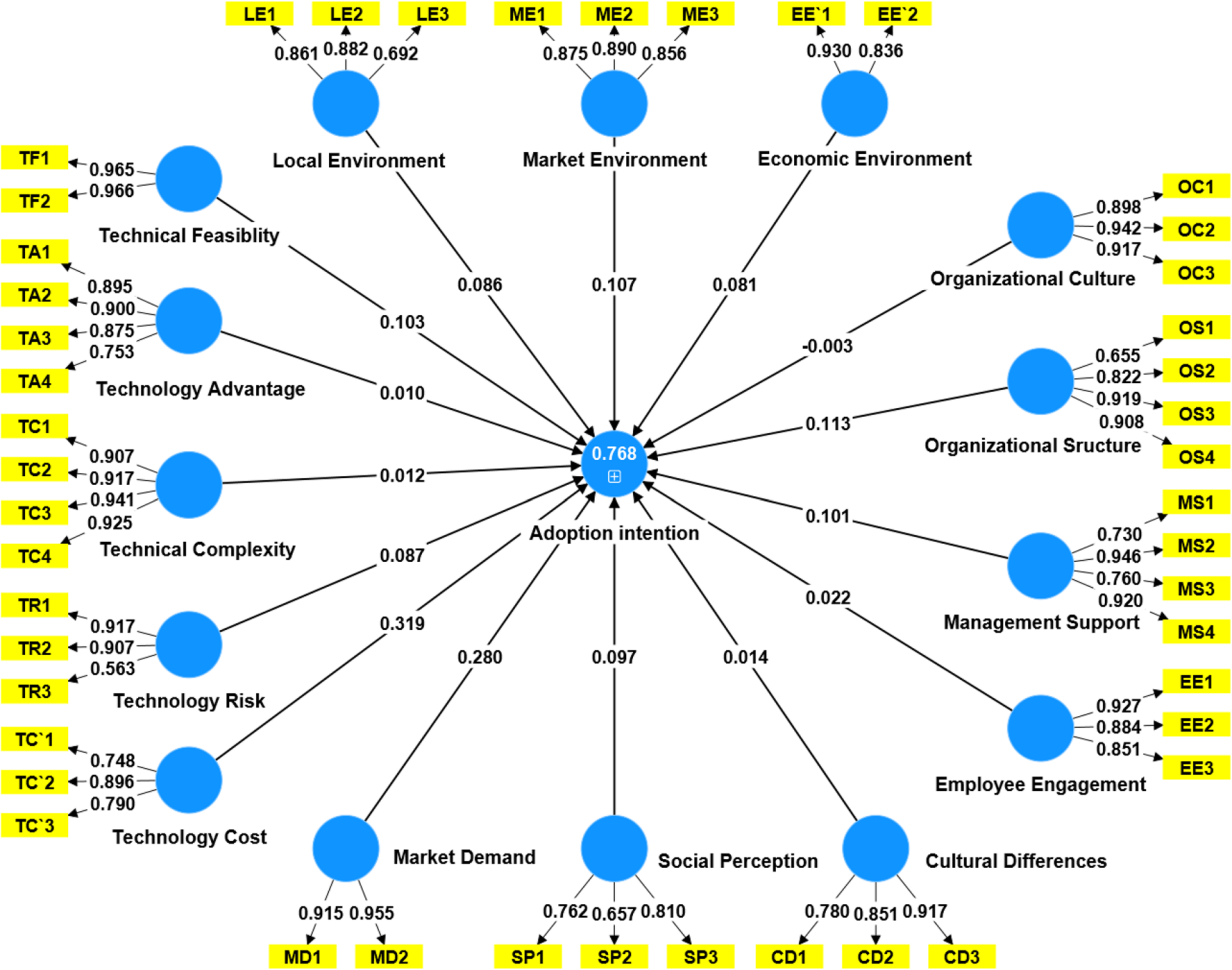
PLS-SEM of adoption intention.

The collected questionnaire data was imported into SmartPLS for testing and validation, and a structural model was generated based on the PLS-SEM algorithm, as shown in Fig. 2. The R2 value for the intention of highway construction enterprises to adopt intelligent construction technology in the model is 0.768, indicating that the model can explain the observed differences in the data fairly well and has a high level of predictive accuracy and reliability^67^. The top five factors with the highest external loadings are technological feasibility (0.966), market demand (0.955), managerial support (0.946), organizational culture (0.942), and technological complexity (0.941). The path coefficients reveal the impact on adoption intention, namely, technological cost (0.319), market demand (0.280), organizational structure (0.113), market environment (0.107), and technological feasibility (0.103).

### PLS-SEM model evaluation

The external loadings of the influencing factors in each dimension are all above 0.7, indicating that the reliability of each indicator reaches a satisfactory level^68^. The composite reliabilities (CR) of the model range from 0.789 to 0.965, which is higher than the recommended threshold of 0.7, indicating that the measurement scales are reliable^69^. As the assessment of convergent validity is based on the average extracted variance (AVE) values, and the computed results in this model are all higher than the recommended threshold of 0.5^70^, the model's construction appears reasonable. The composite reliability of the reflective structure of the model is good, as shown in Table 9.

**Table 9:** The estimation of original external weights of indicators.

Structural path	External model loadings	Composite reliability (CR)	Ave
Technical feasibility	0.965	0.965	0.932
Technology advantage	0.856	0.917	0.736
Technical complexity	0.922	0.958	0.852
Technology risk	0.796	0.848	0.660
Technology costs	0.811	0.854	0.662
Organizational culture	0.919	0.942	0.845
Organizational structure	0.826	0.899	0.693
Management support	0.839	0.907	0.713
Employee engagement	0.887	0.918	0.789
Local environment	0.872	0.856	0.666
Market environment	0.874	0.907	0.764
Economic environment	0.901	0.877	0.782
Market demand	0.935	0.933	0.874
Social perception	0.743	0.789	0.556
Cultural differences	0.849	0.887	0.724
Adoption intention	0.756	0.905	0.581

In order to evaluate the correlation, stability, and accuracy of the model's path coefficients and structural coefficients, and determine which influencing factors in the structural model have a significant impact on the adoption intention, this study further conducted an algorithm evaluation using Bootstrap resampling^69^. After calculating the original external weight estimates, t-values, and corresponding significance p-values of the indicators, the measurement results of the technological, organizational, environmental, and social dimensions are summarized in Table 10. Among them, the p-values of technological risk, technological cost, and organizational culture are all greater than 0.05. However, since these two variables were assumed to have negative impacts, these results can be accepted, indicating that the hypotheses are supported. On the other hand, the hypothesis regarding the positive impact of organizational culture does not hold, thus rejecting hypothesis H2a.

**Table 10:** The estimation of original external weights of indicators.

Structural path	Standard deviation (STDEV)	T-statistic (|O/STDEV|)	p-value	Validation results
Technical feasibility → willingness to adopt	0.054	1.894	0	Support
Technology advantage → willingness to adopt	0.059	0.168	0.038	Support
Technical complexity → willingness to adopt	0.05	0.236	0	Support
Technology risk → willingness to adopt	0.053	1.657	0.052	Support
Technology costs → willingness to adopt	0.046	6.984	0.161	Support
Organizational culture → willingness to adopt	0.055	0.063	0.116	Not supported
Organizational structure → willingness to adopt	0.058	1.942	0	Support
Management support → willingness to adopt	0.055	1.85	0.042	Support
Employee engagement → willingness to adopt	0.06	0.369	0	Support
Policy environment → willingness to adopt	0.04	2.115	0	Support
Market Environment → Willingness to adopt	0.054	1.99	0	Support
Economic environment → willingness to adopt	0.053	1.527	0	Support
Market demand → willingness to adopt	0.045	6.176	0	Support
Social perception → willingness to adopt	0.041	0.33	0	Support
Cultural differences → willingness to adopt	0.049	1.983	0	Support

To further examine the similarity of different indicators in the measurement model, this study used the Heterotrait-Monotrait Ratio (HTMT) standard to evaluate discriminant validity. The results showed that the HTMT ratio of all variables was less than 0.9, indicating that there was not too much overlap between the indicators measuring different latent variables and that the discriminant validity of the scale was high. This is shown in Table 11.

**Table 11:** HTMT results.

Factor	CD	EE`	EE	MS	MD	ME	OC	OS
CD								
EE`	0.68							
EE	0.765	0.578						
MS	0.808	0.626	0.73					
MD	0.83	0.648	0.586	0.612				
ME	0.818	0.661	0.851	0.844	0.727			
OC	0.819	0.583	0.873	0.74	0.604	0.833		
OS	0.862	0.717	0.839	0.834	0.838	0.926	0.852	
LE	0.649	0.603	0.587	0.578	0.629	0.724	0.652	0.658
SP	0.844	0.768	0.754	0.847	0.71	0.821	0.775	0.814
TC	0.829	0.868	0.827	0.801	0.736	0.826	0.816	0.843
TF	0.663	0.653	0.592	0.614	0.633	0.749	0.67	0.696
TA	0.803	0.592	0.725	0.859	0.644	0.825	0.722	0.822
TC`	0.767	0.592	0.795	0.841	0.638	0.813	0.731	0.834
TR	0.849	0.671	0.654	0.749	0.834	0.835	0.673	0.792

Factor	PE	SP	TC	TF	TA	TC`	TR	WA
CD								
EE`								
EE								
MS								
MD								
ME								
OC								
OS								
LE	0.807							
SP	0.634	0.848						
TC	0.877	0.82	0.676					
TF	0.563	0.851	0.826	0.609				
TA	0.635	0.816	0.843	0.613	0.81			
TC`	0.651	0.81	0.795	0.649	0.767	0.694		
TR	0.803	0.825	0.837	0.86	0.821	0.806	0.757	

Secondly, the collinearity between the indicators was evaluated using the Variance Inflation Factor (VIF). Table 12 shows the VIF values of all formative model structures in the model, with the highest being technological cost at 4.154 and the lowest being social cognition at 1.217. All values are below the threshold of 5 .

**Table 12:** Results of collinearity evaluation.

Dimension	Structural path	VIF
Technical dimension	Technical feasibility	3.946
	Technology advantage	2.589
	Technical complexity	4.154
	Technology risk	2.120
	Technology costs	2.035
Organizational dimension	Organizational culture	3.162
	Organizational structure	2.656
	Management support	3.200
	Employee engagement	2.616
Environmental dimension	Local environment	2.024
	Market environment	2.211
	Economic environment	1.496
Social dimension	Market demand	2.313
	Social perception	1.217
	Cultural differences	2.067

Based on the above research, a structural equation model is established as follows:

$$ \begin{aligned} & {\text{Adoption intention}}\, \\ &\quad= \,0.{1}0{9 }*{\text{ technological feasibility}}\, + \,0.0{12 }*{\text{ technological advantage}}\, \\ &\quad\quad+ \,0.0{12 }*{\text{ technological complexity}}\, + \,0.0{9 }*{\text{ technological risk}} - 0.0{11 }*{\text{ technological cost}}\\ &\quad\quad - 0.00{5 }*{\text{ organizational culture}}\, + \,0.{113 }*{\text{ organizational structure}} - 0.{1}0{1 }*{\text{ management support}}\, \\ &\quad\quad + \,0.{32 }*{\text{ employee participation}}\, + \,0.0{84 }*{\text{ local environment}}\, + \,0.{1}0{8 }*{\text{ market environment}}\\ &\quad\quad - 0.0{83 }*{\text{ economic environment}}\, + \,0.{28 }*{\text{ market demand}}\, + \,0.0{99 }*{\text{ social cognition}}\, \\ &\quad\quad + \,0.0{14 }*{\text{ cultural differences}}. \end{aligned} $$

### ANN analysis

Neural networks are computational models that simulate the network of neurons in the human brain^71^. Due to their ability to explore nonlinear relationships between independent and dependent variables, they have been widely applied in data analysis, technology adoption, and predictive modeling. Therefore, this study relied on IBM SPSS 27 software to perform Artificial Neural Network (ANN) predictive analysis. Since the data sample size is large, the Multilayer Perceptron (MLP) algorithm was chosen to train the neural network^72^. The neural network in this study consists of four layers: an input layer, two hidden layers, and an output layer. The input layer consists of 15 independent variables, the output layer represents the dependent variable, which is the willingness of highway construction enterprises to adopt intelligent construction technologies, and the hidden neurons are automatically generated by the SPSS software. The role of the hidden layer nodes is to capture the nonlinear relationships between the input data. The connection weights between the input layer and the first hidden layer, as well as between the two hidden layers, are obtained through model training and learning. The model updates the weight values iteratively to improve the accuracy of predicting the adoption willingness. After training, the model can parse the nonlinear mapping relationship between the input layer data and the output values.

**Figure 3:**
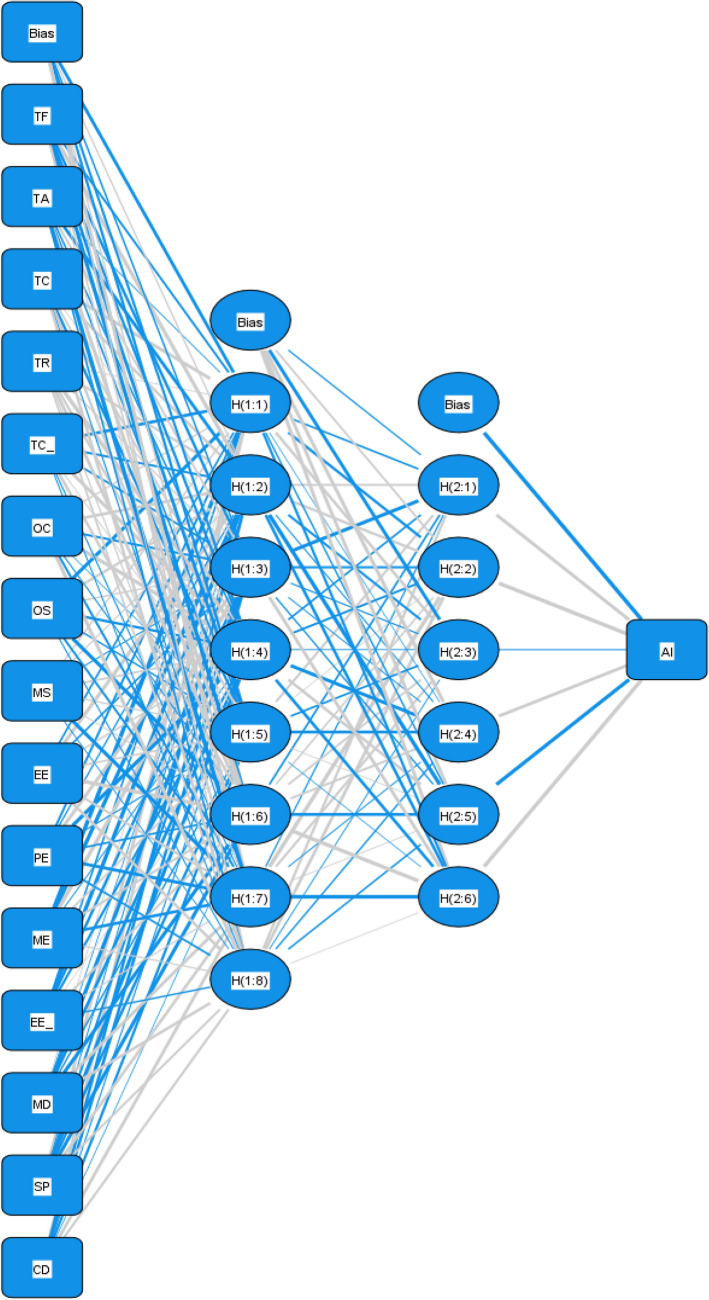
Artificial neural network model diagram.

In addition, to assess the predictive capability of each independent variable, this study conducted a sensitivity analysis by changing one independent variable value while fixing other variable values to evaluate its impact on the output values. This study ranked the relative importance of each independent variable's influence on the output values, as shown in Table 13^73^. Figure 2 shows the artificial neural network model constructed in this study. The sensitivity analysis quantifies the predictive capability of each independent variable. As shown in Fig. 3, this study constructed an artificial neural network model.

**Table 13:** Ranking of relative importance of influencing factors.

ANN	TF	TA	TC	TR	TC`	OC	OS	MS	EE	LE	ME	EE`	MD	SP	CD
1	0.06	0.019	0.072	0.033	0.262	0.036	0.041	0.035	0.025	0.081	0.021	0.039	0.174	0.076	0.026
2	0.034	0.039	0.011	0.017	0.316	0.037	0.124	0.066	0.056	0.037	0.064	0.029	0.122	0.033	0.015
3	0.021	0.018	0.026	0.044	0.266	0.029	0.109	0.062	0.046	0.052	0.056	0.022	0.154	0.076	0.018
4	0.094	0.023	0.023	0.061	0.263	0.021	0.104	0.035	0.02	0.077	0.037	0.028	0.136	0.04	0.037
5	0.059	0.013	0.014	0.021	0.225	0.035	0.091	0.053	0.04	0.128	0.047	0.02	0.158	0.069	0.027
6	0.048	0.015	0.011	0.035	0.26	0.044	0.069	0.05	0.033	0.051	0.135	0.019	0.111	0.09	0.028
7	0.039	0.027	0.023	0.026	0.262	0.025	0.07	0.06	0.085	0.056	0.14	0.033	0.097	0.016	0.041
8	0.089	0.048	0.035	0.075	0.279	0.015	0.022	0.025	0.016	0.08	0.06	0.021	0.158	0.068	0.008
9	0.081	0.011	0.022	0.028	0.23	0.048	0.065	0.03	0.042	0.023	0.148	0.038	0.191	0.024	0.019
10	0.062	0.02	0.012	0.069	0.235	0.017	0.105	0.075	0.023	0.052	0.089	0.023	0.155	0.043	0.021
Mean relative importance	0.059	0.023	0.025	0.041	0.260	0.031	0.080	0.049	0.039	0.064	0.080	0.027	0.146	0.054	0.024
Normalized importance (%)	22.59	8.97	9.58	15.74	100.00	11.82	30.79	18.90	14.86	24.52	30.68	10.47	56.04	20.59	9.24
Ranking	6	15	13	9	1	11	3	8	10	5	4	12	2	7	14

Finally, this study compared and analyzed the ranking results of PLS-SEM and ANN, as shown in Table 14. In the "Match" column of the table, we found that only the rankings of five influencing factors remained unchanged. This may be because PLS-SEM captures the direct linear relationship between variables and extracts principal component information, while ANN analyzes the nonlinear relationship between variables based on the activation levels or weights of neurons. The resulting differences need to be distinguished according to the characteristics of the problem and the nature of the data. Among them, the rankings of Local Environment (LE) and Management Support (MS) have increased significantly, while the rankings of Technology Advantage (TA) and Cultural Differences (CD) have decreased significantly. The rankings of other influencing factors have only changed slightly, which also proves that the two-stage analysis method combining PLS-SEM and ANN used in this study has high value.

**Table 14:** Comparison of PLS-SEM and ANN results.

Variable	Path coefficient	Ranking	Result	Importance (%)	Ranking	Matched
TF	0.103	5	Established	22.59	6	–
TA	0.01	11	Established	8.97	15	–
TC	0.319	13	Established	9.58	13	Matched
TR	0.087	7	Established	15.74	9	–
TC`	−0.012	1	Established	100.00	1	Matched
OC	−0.003	12	Not established	11.82	11	–
OS	0.113	3	Established	30.79	3	Matched
MS	−0.101	15	Established	18.90	8	–
EE	0.022	9	Established	14.86	10	–
LE	0.086	8	Established	24.52	5	–
ME	0.107	4	Established	30.68	4	Matched
EE`	−0.081	14	Established	10.47	12	–
MD	0.28	2	Established	56.04	2	Matched
SP	0.097	6	Established	20.59	7	–
CD	0.014	10	Established	9.24	14	–

### Analysis and results

According to the above PLS-SEM and ANN model calculation results, the path coefficient results of the internal model show that the cost of technology (TC') has the greatest negative influence on the adoption intention, and the path coefficient is -0.319. This is followed by the positive influence of market demand (MD) with a path coefficient of 0.280. Again there is the positive effect of the organizational structure (OS) with a path coefficient of 0.113. According to Table 10, the t-values and p-values of each path are given, and we find that the t-values of technology cost (TC'), market demand (MD) and organizational structure (OS) are large (6.984, 6.176 and 1.942) and the p-values are small (0.161, 0 and 0), indicating that the influence of these three variables on adoption intention is statistically significant. According to the VIF values of each variable in Table 12, the maximum VIF of technology cost (TC') is 4.154, market demand (MD) is 2.313, and organizational structure (OS) is 2.656, indicating that their collinearity is not significant.

In order to avoid subjective and arbitrary judgments and make the conclusions more convincing, this section compares and analyzes the Spearman correlation coefficient, p-value and Kendall correlation coefficient of each influencing factor. It can be seen that there is high consistency between PLS-SEM and ANN in the judgment of the importance ranking of influencing factors, as shown in Table 15.

**Table 15:** Comparison of Spearman coefficient, p-value and Kendall coefficient results.

Structural Path	PLS-SEM ranking	ANN ranking	Spearman coefficient	P-value	Kendall coefficient
Technical feasibility	5	6	0.429	0.024	0.333
Technology advantage	11	15	0.786	0.002	0.733
Technical complexity	13	13	1	0	1
Technology risk	7	9	0.714	0.006	0.6
Technology costs	1	1	1	0	1
Organizational culture	12	11	0.786	0.002	0.733
Organizational structure	3	3	1	0	1
Management support	15	8	0.643	0.01	0.533
Employee engagement	9	10	0.643	0.01	0.533
Local environment	8	5	0.714	0.006	0.6
Market environment	4	4	1	0	1
Economic environment	14	12	0.786	0.002	0.733
Market demand	2	2	1	0	1
Social perception	6	7	0.714	0.006	0.6
Cultural differences	10	14	0.786	0.002	0.733

Specifically, the Spearman correlation coefficient and Kendall correlation coefficient of seven factors, such as technology cost, market demand, organizational structure, market environment, technical complexity, economic environment, and cultural differences, were both 1, and the p-value was 0, indicating that the PLS-SEM ranking of these factors was basically consistent with the ANN ranking. In addition, the Spearman correlation coefficient and Kendall correlation coefficient of technical risk, management support, employee participation, social cognition and other factors were also high, between 0.6–0.8, and the p-value was less than 0.01, indicating that the PLS-SEM ranking of these factors had a significant positive correlation with the ANN ranking. Finally, the correlation coefficient between technical feasibility and organizational culture was lower, but it was still greater than 0.4, and the p-value was less than 0.05, which also indicated that the PLS-SEM ranking of these two factors had a significant positive correlation with the ANN ranking.

On the whole, the ranking correlation test results of each factor are good, indicating that although the PLS-SEM and ANN algorithms are different, the judgment of the importance ranking of the influencing factors is consistent, and the results are reliable. This also confirms that the combination of the two in this study is reasonable and effective. Taking into account all indicators, including correlation coefficients, p-values, and absolute values of rankings, it can be considered that cost of technology (TC'), market demand (MD) and organizational structure (OS) are the three variables that have the greatest impact on adoption intentions, and should be among the top three.

## Discussion

According to the results of the PLS-SEM and ANN models, except for Organizational Culture, which has no significant effect on the willingness to adopt, all other hypotheses have been validated and have significant effects on the willingness of Chinese highway construction enterprises to adopt intelligent construction technology. The study emphasizes some important determining factors, with Technology Costs (TC), Market Demand (MD), Organizational Structure (OS), and Market Environment (ME) considered the top four. These results enrich the existing literature on the adoption of intelligent construction technology in highway construction enterprises and help practitioners and policymakers better understand the willingness of construction enterprises to adopt blockchain technology. The specific research results are as follows:

1. In terms of the technical dimension, assuming H1a is validated, i.e., the feasibility of the technology has a significant positive impact on the adoption intention, which is consistent with the findings of scholars such as Nguyen, Franco, Sarker, and Warren^74–77^. The assessment of the technical feasibility of intelligent construction technology can help highway construction enterprises better understand the technical difficulties, bottlenecks, and potential issues behind new technologies, provide practical data and information support to enterprises, facilitate comprehensive evaluation of investment risks, and achieve reasonable returns, enabling more accurate decision-making. Assuming H1b is established, i.e., the technological advantage has a significant positive impact on the adoption intention, which is consistent with the views of scholars such as Alamo, Wang, and Zhao^78–80^. Technological advantages can enhance a company's competitiveness and drive continuous innovation. When highway construction enterprises adopt intelligent construction technologies with obvious advantages, they can effectively improve work efficiency, reduce costs, and enhance engineering quality, thus strengthening their competitive advantages over their peers and continuously improving their core competitiveness through technological innovation and upgrades. Assuming H1c is established, i.e., the complexity of the technology has a significant negative impact on the adoption intention, which is consistent with the research results of scholars such as Hadwer, Janssen, Oyelaran, and Parvand^81–84^. Intelligent construction technology is relatively complex compared to traditional construction techniques, and its adoption involves higher risks, requiring the application of various skills and tools, as well as more training or complete contingency measures. Therefore, highway enterprises may adopt a wait-and-see attitude towards intelligent construction technologies with high technical complexity. Assuming H1d is established, i.e., the technological risk has a significant negative impact on the adoption intention, which is consistent with the findings of scholars such as Ghaffarianhoseini, Janssen, and Zhao^15,80,82^. The technological risks of adopting intelligent construction technologies include safety risks, compliance risks, and data risks, among others. As intelligent construction technologies require processing a large amount of data, if the data quality is poor or mishandled, it may lead to information leakage. The implementation of intelligent construction technologies may involve legal, regulatory, and standard compliance issues, and if enterprises violate relevant laws and regulations, they will face financial and reputational risks. Unverified or incorrect design and construction plans may increase the risk of major accidents, posing potential threats to building and worker safety. Assuming H1e is established, the technological cost has a significant negative impact on the adoption intention, this factor has been found to have the strongest negative impact on the adoption intention in this study, which is consistent with the research by Liu, Zakeri, and Zhao^80,85,86^. Technological costs include not only the current investment costs for hardware, software, human resources, and training but also the uncertainty costs associated with the development and application due to the wide range of application, technical complexity, and numerous areas involved. Therefore, to promote the widespread application of intelligent construction technology, highway construction enterprises need to comprehensively consider factors such as technology cost investment, types of technology, and long-term return on investment. They should continuously explore and adopt more effective methods to reduce technology costs and risks, thus improving the effectiveness and benefits of using intelligent construction technology.
2. In terms of organizational dimensions, except for the hypothesis H2a, hypotheses H2b, H2c, and H2d are all supported. This means that organizational structure, management support, and employee involvement have a significant positive impact on the willingness to adopt intelligent construction technology, which is consistent with the findings of scholars such as Baduge, Maddikunta, Maroufkhani, Pan, and Tavallaei^87–91^. The study shows that innovation and friendly organizational structures (such as horizontal structures, cross-functional teams, and open communication channels) facilitate the adoption and application of intelligent construction technology. With management support, employees are more likely to accept and use intelligent construction technology and allocate sufficient resources to promote its application. As for employee involvement, active participation by employees helps optimize technology implementation plans, improve the effectiveness of technology use, and enhance management levels. Therefore, the higher the employee involvement during the adoption of intelligent construction technology, the greater the willingness of the enterprise to adopt it.
3. The hypotheses H3a, H3b, and H3c regarding local environment, market environment, and economic environment have all been verified, which aligns with the perspectives of scholars such as Asadi, Cao, Hong, Kothari, Mhatre, and Woerter^37,92–96^. Policy support and industry norms in the local environment are essential factors influencing adoption. Some countries and regions have implemented policies to promote the application of BIM and intelligent construction technology in the construction industry, for example, China's "Smart Construction Site" policy. Industry support and norms from governments and local authorities can help highway construction enterprises more easily accept and apply intelligent technology. Influenced by industry trends, technological changes, and market scale growth, highway construction enterprises must timely adjust and optimize their own technological levels and operational modes, strengthen the stability of cooperative relationships with suppliers in order to better adapt to the market environment and win market share. In the current economic environment, cost reduction, time saving, and efficiency improvement are crucial for enterprises. Intelligent construction technology not only improves engineering quality but also reduces construction costs and time. Moreover, due to the significant investment required for intelligent construction technology, the investment environment directly affects whether enterprises adopt these technologies.
4. Hypotheses H4a, H4b, and H4c in the social dimension have all been validated. Specifically, H4a states that market demand and social cognition have a significant positive impact on the willingness to adopt intelligent construction technology, while cultural differences have a significant negative impact. This is consistent with the findings of scholars such as Balta-Ozkan, Frustaci, Liu, Mao, and Wu^97–101^. Market demand determines the scope of application, research and development investment, market prospects, and commercialization of intelligent construction technology in the highway construction field. An increase in market demand attracts more highway construction investments in the research and development of intelligent construction technology to meet the market's demand for better, more convenient, and more efficient construction services. Additionally, an increase in market demand brings about more business opportunities and market share, driving the commercialization of intelligent construction technology and enhancing its competitiveness and market share. Some highway construction enterprises may lack practical experience or technical capabilities in the actual application of intelligent construction technology, leading to certain resistance towards its commercialization. Relevant studies have shown that intelligent construction technology relies on digitalization, automation, and other means, which may require traditional construction enterprises to undergo technological transformation and adaptation to acquire operational and practical experience with new technologies. Therefore, it is crucial to enhance recognition and willingness to adopt intelligent construction technology within the construction industry. Cultural differences can influence people's perception of the trustworthiness and safety of intelligent construction technology. Research has shown that individuals from certain cultural backgrounds may be more sensitive to the security and privacy protection of technology, which can affect their trust and willingness to adopt intelligent construction technology. For example, in European and American countries, there is a higher sensitivity to privacy issues such as data protection, which may impact their acceptance and willingness to adopt intelligent construction technology.

## Concluding remarks

Based on the existing theories and literature, this study incorporates social factors into the type, proposes the TOSE framework, and formulates four dimensions and 15 factors affecting the willingness of China's highway construction enterprises to adopt intelligent construction technology by combining the results of expert interviews, and then collects 513 valid data through a questionnaire survey. Then, 513 valid data were collected through a questionnaire survey, and the linear and non-linear relationships and importance of factors affecting willingness to adopt were analyzed using a combination of PLS-SEM and ANN. Except for organizational culture, all other 14 factors had varying degrees of promotion or hindrance effects on willingness to adopt. Among the influencing factors in the technology dimension, the impact of technology cost was the strongest, which is consistent with the research findings of previous scholars and also indicates the authenticity and reliability of the results of this study.In terms of practical implications, the results of this study can be used as a reference for government departments in formulating policies to encourage the adoption of technology in construction firms, and it can also help business managers to better evaluate and make adoption decisions. In terms of social implications, this study can promote the idea that the adoption of smart building technologies can increase productivity and safety in the construction industry, thereby benefiting society as a whole, and it can also be promoted through popular science articles and other forms of dissemination in order to influence the public's perceptions and attitudes towards new technologies. And wider adoption of advanced technologies can indirectly improve the quality of life by enhancing infrastructure and reducing environmental impacts. In teaching and learning, the findings of this study can also provide cases for related courses. The findings also new research perspectives will also enrich relevant theories in the field of technology management. The added insights of sample representativeness, expansion of research perspectives, practical applications, and theoretical contributions enrich and complete the research implications of the article.

In addition, this study may have some limitations. Since this study used cross-sectional data, it may not reflect dynamic changes, and future studies may consider adopting a long-term tracking research design to capture the dynamic changes in the firms' adoption process more comprehensively. Second, the sample of this study is limited to highway construction, and other construction firms may have different influencing factors. Therefore, future research could expand the sample to include other types of construction firms to improve the generalizability of the findings. Meanwhile, this study mainly focuses on the influencing factors at the technical level, and future studies may consider further exploring the adoption behavior from other perspectives such as management and culture, and comparisons with firms in different countries and regions will also provide valuable insights.

Regarding the issue of technology cost, future research may focus on ways and strategies to reduce technology costs. In addition to reducing technology costs through technological innovation and policy support, there may be other feasible ways to reduce technology costs, such as adopting a sharing economy model and promoting industrial collaboration. In terms of the organizational dimension, future research should focus on the coordination of internal organizational structure and external market environment of enterprises. The willingness and level of adoption of intelligent construction technology by enterprises are not only related to the attributes of the technology itself but also to factors such as the organizational structure and management methods of the enterprise. Therefore, future research needs to explore the impact mechanism and decision-making process of internal organizational change on the adoption of intelligent construction technology by enterprises. Local governments play an important role in policy support, management, and market guidance of intelligent construction technology, which directly affects the willingness and degree of adoption of intelligent construction technology by enterprises. Therefore, it is necessary to study the role mechanism, policy formulation, and management methods of local governments in the promotion and application of intelligent construction technology. In terms of the social dimension, the changing needs and attitudes of stakeholders such as collaborative enterprises and the public should be taken into account. With the continuous improvement of awareness, changes in demand for products and services will also occur, and it is necessary to grasp the mechanism and trends of demand changes.

In summary, in the future, we should adhere to the principles of openness, innovation, and collaboration, and conduct in-depth research on the willingness and promotion of highway construction enterprises to adopt intelligent construction technology from multiple perspectives such as technology, organization, environment, and society, continuously explore and innovatively apply technological means, and provide more comprehensive, scientific, and effective support for the application and development of intelligent construction technology, and to further explore the culture of adopting behaviors and the management perspectives for the future research.

### Supplementary Information

Supplementary Information 1.

Supplementary Information 2.

Supplementary Information 3.

## Data Availability

The data generated and analyzed during the current study are not publicly available due to the ethics approval that promised participants to only use the data for academic research purposes and maintain absolute confidentiality of any confidential/sensitive information that may identify or harm the participants. Thus the research data cannot be made public in order to protect the privacy of the participants. However, the dataset can be obtained from the corresponding author on reasonable request whilst this research is ongoing.
