# Supplementary material for: Analysis of factors of willingness to adopt intelligent construction technology in highway construction enterprises
Source: Sci Rep. 2023 Nov 7;13:19339. doi: 10.1038/s41598-023-46241-6 (PMC10630408; doi:10.1038/s41598-023-46241-6)
Supplement: Supplementary file 3 — Supplementary Information 3. [file 41598_2023_46241_MOESM3_ESM.docx]

Appendix 1

Dear Sir/Madam,

Thank you very much for participating in this academic questionnaire survey. With the advent of the digital construction era, intelligent construction technology has gradually attracted the attention and application of experts and scholars at home and abroad. This research project is conducted by the School of Civil Engineering at Northeast Forestry University, aiming to explore the important influencing factors that hinder the adoption of intelligent construction technology by highway construction enterprises. This survey questionnaire is filled out anonymously. Please fill it according to your actual situation. Your answers are not right or wrong, and represent your personal views. The survey results of this questionnaire are only used for academic research and will not be used for any commercial purposes.

We will strictly keep confidential any information about your personal information, and the analysis results of the data are only for the use of this study.

School of Civil Engineering and Transportation, Northeast Forestry University.

**Part One: Personal Basic Information (Please mark "√" on the appropriate options)**

1. Your educational background:

□ Junior college or below

□ Undergraduate

□ Master's degree

□ Doctoral degree or post-doctoral research

2. Your years of work experience:

□ 5 years or less

□ 5-10 years

□ 10-15 years

□ 15 years or more

3. Your current working unit:

□ Design unit

□ Construction unit

□ Higher education institution

4. Your current position:

□ General manager

□ Project manager

□ Chief engineer

□ Departmental manager

□ Professor or associate professor

5. Your understanding of intelligent construction technology:

□ Very familiar

□ Familiar

□ Not very clear

□ Never heard of it

**Part Two: Research Questions**

Please fill in the numbers according to your understanding and experience of our company and intelligent construction technology. Please choose an integer between 1-5. "1" means "strongly disagree," "2" means "disagree," "3" means "uncertain," "4" means "agree," and "5" means "strongly agree." There is no right or wrong answer, please fill it according to your own situation.

(Note: The following content is a questionnaire, which cannot be translated without more information about the questions being asked.)

| Dimension | Influencing factors | Research questions | Scoring |
| --- | --- | --- | --- |
| Technology Dimension | Technical Feasibility | TF1: Do you think the adoption of intelligent construction technology can reduce construction difficulty? |  |
|  |  | TF2: Do you think that intelligent construction technology is more applicable than traditional technology in the process of highway construction? |  |
|  | Technology Advantage | TA1: Do you think that the adoption of intelligent construction technology can reduce environmental pollution and improve construction safety? |  |
|  |  | TA2: Do you think that the adoption of intelligent construction technology is helpful for design and construction management? |  |
|  |  | TA3: Do you think that the adoption of intelligent construction technology is helpful for improving operational and management efficiency? |  |
|  |  | TA4: Do you think that the adoption of intelligent construction technology can bring practical benefits to the enterprise? |  |
|  | Technical Complexity | TC1: The interviewee believes that intelligent construction technology is very complex. |  |
|  |  | TC2: The interviewee believes that learning intelligent construction technology requires a great deal of effort. |  |
|  |  | TC3: The interviewee believes that applying intelligent construction technology requires sufficient experience. |  |
|  |  | TC4: The interviewee believes that the application of intelligent construction technology is a complex process. |  |
|  | Technology Risk | TR1: The interviewee thinks that there will be new safety hazards when applying intelligent construction technology. |  |
|  |  | TR2: The interviewee thinks that applying intelligent construction technology may cause information or data leakage. |  |
|  |  | TR3: The interviewee thinks that there may be vulnerabilities or security issues in the storage or processing of intelligent construction technology. |  |
|  | Technology Costs | TC`1: The interviewee believes that the cost of applying intelligent construction technology is not high. |  |
|  |  | TC`2: The interviewee believes that applying intelligent construction technology will not increase the cost of purchasing, operating, and maintaining new hardware or software. |  |
|  |  | TC`3: The interviewee thinks that the training cost for intelligent construction technology is not high. |  |
| Organizational Dimension | Organizational Culture | OC1: The interviewee's company focuses on the development and application of new technologies. |  |
|  |  | OC2: The interviewee's company often conducts technological innovation or company development exchanges. |  |
|  |  | OC3: The interviewee's company has smooth communication within and outside the company, with a strong sense of trust. |  |
|  | Organizational Structure | OS1: The interviewee's company has a flexible organizational structure and smooth information flow. |  |
|  |  | OS2: Decision-making efficiency in the interviewee's company is high. |  |
|  |  | OS3: Command and execution in the interviewee's company are orderly. |  |
|  |  | OS4: Each department in the interviewee's company works closely together. |  |
|  | Management Support | MS1: The top management of your company provides sufficient support for adopting intelligent construction technology. |  |
|  |  | MS2: The top management of your company is willing to take risks in adopting and applying intelligent construction technology. |  |
|  |  | MS3: The top management of your company believes that adopting intelligent construction technology will contribute to the future development of the enterprise. |  |
|  |  | MS4: The top management of your company has provided ample resources for adopting intelligent construction technology. |  |
|  | Employee Engagement | EE1: The employees of your company support the adoption of intelligent construction technology. |  |
|  |  | EE2: The employees of your company are willing to participate in the training and learning of intelligent construction technology. |  |
|  |  | EE3: The employees of your company often discuss intelligent construction technology. |  |
| Environmental Dimension | Local Environment | LE1: The government provides policy and funding support for enterprises that adopt intelligent construction technology. |  |
|  |  | LE2: The government encourages highway construction companies to adopt intelligent construction technology. |  |
|  |  | LE3: The government has detailed planning for the adoption of intelligent construction technology by highway enterprises. |  |
|  | Market Environment | ME1: Your company believes that adopting intelligent construction technology will bring market competitive advantages. |  |
|  |  | ME2: Some of your company's competitors have already begun to apply intelligent construction technology. |  |
|  |  | ME3: Your company believes that if it does not adopt intelligent construction technology, it will be at a disadvantage in the future market. |  |
|  | Economic Environment | EE`1: Your company believes that if the economic benefits are good enough, it will adopt intelligent construction technology. |  |
|  |  | EE`2: Your company believes that it will adopt intelligent construction technology when labor costs are low. |  |
| Social Dimension | Market Demand | MD1: Your company believes that it will adopt intelligent construction technology when there is high market demand. |  |
|  |  | MD2: Your company believes that it will adopt intelligent construction technology when investment and tax environments are favorable. |  |
|  | Social Perception | SP1: If your company realizes the practical application and potential value of intelligent construction technology, it would tend to adopt it. |  |
|  |  | SP2: Your company is able to make basic judgments about the development after the adoption of intelligent construction technology. |  |
|  |  | SP3: Your company has a strong willingness to adopt intelligent construction technology. |  |
|  | Cultural Differences | CD1: Your company believes that communication and coordination difficulties between different cultural regions will affect the adoption of intelligent construction technology. |  |
|  |  | CD2: Your company believes that there are significant differences in understanding and willingness to adopt intelligent construction technology among different cultural regions. |  |
|  |  | CD3: Your company believes that employees from different cultural regions should strengthen team communication, avoid cultural conflicts and misunderstandings after adopting intelligent construction technology. |  |

**Conclusion**

The survey has now concluded, and we would like to express our gratitude for your support in this research study!

If you are interested in this study, please leave your contact information. We will be more than happy to share the research results with you once the study is completed.

Contact Person: E-mail:
